# Effect of *Bacillus subtilis* C-3102 Spores as a Probiotic Feed Supplement on Growth Performance, Nutrient Digestibility, Diarrhea Score, Intestinal Microbiota, and Excreta Odor Contents in Weanling Piglets

**DOI:** 10.3390/ani12030316

**Published:** 2022-01-27

**Authors:** Jing Hu, In-Ho Kim

**Affiliations:** 1 College of Life Science, Linyi University, Shuang Ling Road Middle Section, Linyi 276400, China; ruanjingyi314@163.com; 2 Department of Animal Resource & Science, Dankook University, Cheonan 330-714, Choongnam, Korea

**Keywords:** *Bacillus subtilis*, growth performance, microbiota, nutrient digestibility, weanling piglets

## Abstract

**Simple Summary:**

The weanling period is a vital stage for piglets. Due to lack of a complete digestive system and an immune system, a series of stress problems develop, such as diarrhea. As a kind of microecological additive with high stability and rapid proliferation, *Bacillus* *subtilis* is suitable for piglets’ diets as an additive. The objective of our study was to determine and confirm the effect of *Bacillus* *subtilis* C-3102 spores as a probiotic feed supplement on growth performance, nutrient digestibility, fecal score, intestinal microbiota, and excreta odor contents in weanling piglets. Taken together, our results suggest that dietary supplementation with *Bacillus* *subtilis* C-3102 spores could benefit the body weight, average daily gain, and gain-to-feed ratio of weanling piglets and improve the ATTD of dry matter, crude protein, and energy.

**Abstract:**

It has been well-documented that the dietary supplementation of *Bacillus* *subtilis* could improve piglet performance. The present study was conducted to investigate the effect of *Bacillus subtilis* C-3102 spores as a probiotic feed supplement on growth performance, nutrient digestibility, fecal score, intestinal microbiota, and excreta odor contents in weanling piglets. A total of 150 crossed ((Yorkshire × Landrace) × Duroc) weanling piglets (28-days-old), with an average initial body weight of 7.53 ± 1.23 kg, were divided into two treatment groups according to sex and initial body weight (BW) for a 6-week experiment. In each group, fifteen replicate pens consisting of five piglets per pen (three gilts and two barrows) were used in a randomized complete block design. Treatments consisted of (1) CON, a basal diet; (2) BSC, a diet of CON + 300 g *Bacillus subtilis (B. subtilis)* C-3102 spores per ton of feed. Supplementation with the *B. subtilis* C-3102 spores in the diet increased the BW, average daily gain (ADG), and gain-to-feed ratio (G:F) throughout the whole trial (*p* < 0.05). Weanling piglets that were fed *B. subtilis* C-3102 spores had increased dry matter (DM), crude protein (CP), and energy (E) digestibility compared to the CON group (*p* < 0.05). Lower diarrhea scores were observed in the *B. subtilis* C-3102 spores group on Day 7 (*p* < 0.05). Taken together, our results suggest that dietary supplementation with *B. subtilis* C-3102 spores could benefit the BW, ADG, and G:F of weanling piglets and improve the apparent total tract digestibility (ATTD) of the DM, CP, and E.

## 1. Introduction

The gastrointestinal peristalsis ability of piglets is weak and gradually shows regular changes with aging until they are 60–90 days old, before approaching the level of adult piglets. Additionally, the nutritional, physiological (for example, the immaturity of the mucosal barrier or weanling from breast milk), and environmental stressors have an adverse impact on the health of piglets and, thus, piglets are prone to diarrhea, stagnant growth, and even death in the process of weanling [1,2]. Low gastric acid of piglets lessens the bactericidal properties, allowing pathogenic bacteria to cross the gastric barrier, resulting in poor digestion and absorption of nutrients in piglets. Probiotics can produce various digestive enzymes in animals—such as *Bacillus sp.,* which produces highly active proteases, lipases, and amylases—to reduce certain anti-nutritional factors in feed and degrade some of the more complex carbohydrates in plant feed, thereby improving feed utilization [3,4]. Spores are considered to have stable performance, especially in the process of feed manufacturing and storage [5]. *Bacillus subtilis* is aerobic or facultatively anaerobic. After entering the gastrointestinal tract, spores colonize the gut, consuming a large amount of free oxygen and inhibiting the growth of harmful aerobic bacteria (*Escherichia coli, Streptococcus, Staphylococcus aureus*), which results in an enabling environment for other beneficial bacteria (*Lactobacillus, Bifidobacterium*), that could explain the observed reduction in the incidence of diarrhea in young livestock and poultry [6].

Jeong and Kim (2014) summarized that the main mode of action of *B. subtilis* C-3102 appears to create an anaerobic environment in the intestines after germination, which is conducive to the growth and proliferation of the natural microbial *Lactobacillus*, thereby inhibiting intestinal pathogenic bacteria [7]. *B. subtilis* C-3102 spores have been reported to improve performance and microbiological status in broilers [7,8,9] and laying hens [10], but there has been limited research on its effect on piglets [11,12,13], especially weanling piglets. Therefore, this study was conducted to investigate the effect of *B. subtilis* C-3102 spores as a probiotic feed supplement on growth performance, nutrient digestibility, fecal score, intestinal microbiota, and excreta odor contents in weanling piglets.

## 2. Materials and Methods

All animal-based procedures were done in accordance with the Guidelines for the Care and Use of Experimental Animals of Dankook University (Cheonan, South Korea; approval code: DK-2-1705/2017-02-01).

### 2.1. Bacterial Strain

The final *B. subtilis* C-3102 product, Calsporin^®^, was provided by a commercial company (Calpis Co. Ltd., Tokyo, Japan) and was composed of spray-dried spore-forming *B. subtilis* C-3102 endospores (BSC). The final product was determined to contain at least 1.0 × 10^9^ cfu/g of *B. subtilis* and was kept in a sterilized container before use.

### 2.2. Experimental Design, Animals, and Facilities

A total of 150 crossed ((Yorkshire × Landrace) × Duroc) weanling piglets (28-days-old) with an average initial body weight (BW) of 7.53 ± 1.23 kg were divided into two treatment groups according to sex and initial BW in a 6-week experiment. In each group, fifteen replicate pens consisting of five piglets per pen (three gilts and two barrows) were used in a randomized complete block design. The dietary treatment groups were as follows: (1) CON, basal diet; (2) BSC, CON + 300 mg of BSC/ton of feed. The study was divided into Phase 1 (Day 1–7), Phase 2 (Day 8–21), and Phase 3 (Day 22–42), according to the NRC (2012) recommendations for ((Yorkshire × Landrace) × Duroc) piglets. The diets were formulated to meet or exceed the NRC (2012) nutrient requirements [14]. The piglets were placed in nursery pens (0.6 × 2.0 m × 0.5 m) made of stainless steel and a slatted plastic floor in environmentally controlled rooms. Each pen was equipped with a stainless-steel feeder and a nipple drinker that allowed for ad libitum access to feed and water supply during the trial period. A mechanical system of ventilation was applied, and artificial light was automatically provided regularly for 12 h each day. The indoor temperature was approximately 30 °C, and it decreased by 1 °C each week during the experiment. The ingredients and chemical composition of the total mixed rations are detailed in Table 1.

### 2.3. Sampling and Measurements

At Days 0, 7, 21, and 42, the individual piglets’ body weights and feed disappearance were recorded to determine the average daily gain (ADG), the average daily feed intake (ADFI), and the gain-to-feed ratio (G:F) in piglets. Chromium oxide (Cr_2_O_3_, 2 g/kg) was added to the diets as an indigestible marker during days 36–42. Fresh fecal grab samples were randomly obtained once by rectal massaging from at least two piglets (1 gilt and 1 barrow) in each pen at the end of the experiment to determine the apparent digestibility of dry matter (DM), crude protein (CP), and energy. All samples were stored immediately at −20 °C until analysis. The fecal samples were dried at 70 °C for 72 h and finely ground to pass through a 1-mm screen [16]. The procedures utilized for the determination of DM (method 930.15) and CP (method 920.39) digestibility were conducted in accordance with the methods described by the AOAC (2000) [17]. Chromium was analyzed via UV absorption spectrophotometry (model UV-1201, Shimadzu, Kyoto, Japan) according to the methods of Williams et al. [18]. The apparent total tract digestibility (ATTD) of DM and CP were also calculated using the methods described by Williams et al. [18]. The gross energy (E) was determined by using a Parr 6100 oxygen bomb calorimeter (Parr Instrument Co., Moline, IL, USA).

At Day 42, the fecal samples of one randomly selected piglet (one gilt and one barrow) each were collected from each pen. They were pooled by pen and placed on ice for transport to the analysis laboratory. One gram of fresh sample from each pen was taken and diluted with 9 mL of 1% peptone broth (Becton, Dickinson, and Co.) and homogenized. Viable counts of bacteria in the fecal samples were then determined by plating 10-fold serial dilutions (in 1% peptone solution) onto MacConkey agar plates (Difco Laboratories, Detroit, MI, USA) and lactobacilli medium III agar plates (Medium 638; DSMZ, Braunschweig, Germany) for the isolation of the *Escherichia coli* (*E. coli*) and *Lactobacillus*, respectively. The lactobacilli medium III agar plates were then incubated for 48 h at 39 °C, and the MacConkey agar plates were incubated for 24 h at 37 °C under anaerobic conditions. The *E. coli* and *Lactobacillus* colonies were counted immediately after removal from the incubator. 

At 08.00 and 20.00 h, the diarrhea scores were visually assessed using a subjective score and recorded at Day 0, Day 1-7, Day 15-21, and Day 35-42 by the same person. The diarrhea scores were determined as the average values of five piglets from each pen using a 5-grade score system [19]. The standard of this system is as follows: 1 = hard, dry pellets in a small, hard mass; 2 = firm, formed stool that remains firm and soft; 3 = soft, formed, and moist stool that retains its shape; 4 = loose, unformed stool that assumes the shape of the container; 5 = watery, liquid stool that can be poured. The scores were recorded for each pen following the observations of individual piglets and signs of stool consistency in the pen.

Fecal samples were collected directly from two piglets (one gilt and one barrow) in each pen at the end of the experiment for the determination of excreta odor contents. A total of 300 g of fresh feces from each pen were stored in 2.6 L plastic boxes in replicates. The samples were fermented for 48 h at a room temperature of 32 °C. After the fermentation period, a Gastec (model GV-100) gas sampling pump was utilized for gas detection (Gastec detector tube No. 3M and 3La for ammonia (NH_3_); No. 4LL and 4LK for hydrogen sulfide (H_2_S); No. 70 and 70L for total mercaptans, Gastec Corp., Kanagawa, Japan).

### 2.4. Statistical Analyses

The pen was used as the experiment unit and all data were analyzed with SAS version 9.1 (SAS Institute Inc., Cary, NC, USA) using the mixed general linear model procedure. Differences between treatments were detected by Tukey’s multiple range test. The results are expressed as the least squares means and standard error. A significant difference level of 0.05 was used to determine statistical significance, and a level of 0.10 was considered a trend.

## 3. Results

### 3.1. Growth Performance

The results of growth performance are summarized in Table 2. Supplementation with *B. subtilis* C-3102 spores increased the BW by Day 21 and Day 42 (*p* = 0.002, *p* = 0.003, respectively). The inclusion of *B. subtilis* C-3102 spores as probiotics improved the ADG in the Day 0–7, Day 8–21, and Day 22–42 periods, as well as during the overall experimental period (*p* = 0.044, *p* = 0.029, *p* = 0.014, *p* = 0.004, respectively). Piglets fed *B. subtilis* C-3102 spores showed significantly higher (*p* = 0.027) ADFI by Day 8–21 and the overall experimental periods (*p* = 0.041). The G/F in the overall experimental periods improved significantly (*p* = 0.011) when the piglets were fed *B. subtilis* C-3102 spores.

### 3.2. Nutrient Digestibility

As shown in Table 3, the ATTD of DM in piglets receiving a *B. subtilis* C-3102 spore-supplemented diet was higher (*p* = 0.032) than that in the CON group. Piglets fed a *B. subtilis* C-3102 spore diet exhibited an increased (*p* < 0.0001) ATTD of CP compared to animals fed the CON diet. In addition, the energy digestibility of piglets fed *B. subtilis* C-3102 spores was improved (*p* = 0.0001) compared to the CON group.

### 3.3. Diarrhea Score

Table 4 shows the results of the fecal scores. Piglets that received diets supplemented with *B. subtilis* C-3102 spores had lower (*p* = 0.047) diarrhea scores compared to the non-supplemented piglets at Day 1–7. However, no differences were observed at Days 0, 15–21, or 35–42.

### 3.4. Intestinal Microbiota

As illustrated in Table 5, the concentrations of *E. coli* and *Lactobacillus* tended to decrease and increase, respectively (*p*= 0.063, *p*= 0.076), when piglets were fed a diet supplemented with *B. subtilis* C-3102 spores throughout the entire experimental period compared to the CON group.

### 3.5. Excreta Odor Contents

The results of the excreta odor emissions in piglets are presented in Table 6. The results show that piglets fed with the diet supplementation of *B. subtilis* C-3102 spores tended to have a decrease (*p* = 0.053) in the concentration of fecal total mercaptan emissions compared to the CON group. However, ammonia (NH_3_) and hydrogen sulfide (H_2_S) levels were not affected (*p* > 0.05) by dietary BSC spore supplementation.

## 4. Discussion

As more restrictions are placed on ingredients used in economical animal feed, methods for improving the health performance of piglets by adding additives into feed have received more attention [11]. Weanling piglets often show poor appetites, slow growth, low feed utilization, and diarrhea due to the stress of various factors, such as feed and environment. The beneficial effects of *B. subtilis* supplementation on animal performance have been well-documented in previous studies [2,11]. Our results demonstrated that BSC supplementation significantly increased the BW, the ADG, the ADFI, and the G/F compared to the CON group throughout the whole experiment. The results are in agreement with another study demonstrating highly significant increases in the final BW and ADG compared to the control animals and an improvement in the gain-to-feed ratio in the treated group [20]. Similarly, growth could be promoted when piglets fed diets supplemented with *B. subtilis* C-3102 (Calsporin^®^) [21]. Significant improvements to performance parameters (final BW, ADG, and G:F) were recorded after Calsporin^®^ (*B. subtilis* C-3102) consumption [12]. Marubashi et al. (2012) reported that piglets fed diets supplemented with a commercial probiotic product (Calsporin^®^: *B. subtilis* C-3102) were significantly heavier (3.4%) at 43 days. In addition, Michiels et al. (2016) also reported that the piglets fed diets supplemented with *B. subtilis* C-3102 showed a significant G:F improvement [13]. At present, the mechanism of probiotics that improves animal production performance and promotes growth is not completely understood. Nevertheless, most researchers generally believe that probiotics as feed additives can regulate intestinal flora, promote the digestion and absorption of nutrients, and enhance the immunity of the animal body [22]. In agreement with these results, our study indicated that *B. subtilis* C-3102 spores as probiotics improved the BW, ADG, and G:F in weanling piglets. However, some conflicting results were found by other researchers. Regarding BW, Menegat et al. (2017, 2018) reported no evidence for differences between piglet diets supplemented with a commercial probiotic product (Calsporin^®^: *B. subtilis* C-3102) and those without [5,23]. The different conclusions produced by these studies may be related to the composition of the diet, the age of the piglets, and interactions with environmental factors and dietary feed additives [24].

*Bacillus spp.* can produce a variety of digestive enzymes, such as protease, lipase, and amylase, in the intestinal tracts of animals [3,25]. At the same time, many kinds of nutrients, such as amino acid and growth-promoting factors, were produced by *Bacillus spp.* to promote the metabolisms of animal bodies [26]. Improved digestibility is a key factor in improved performance. In our study, the ATTD of DM, CP, and E were significantly improved by dietary supplementation with *B. subtilis* C-3102 spores. In addition, our results show there were positive effects on the BW, ADG, ADFI, and G/F in the BSC group. In agreement with our study, Kim et al. (2010) reported that the digestibility of DM, CP, and nitrogen was higher in piglets supplemented with bacillus probiotics than in piglets fed a control group diet [27]. In a report by Devi and Kim (2014), the digestibility of DM, N, and E were found to be increased significantly with a *B. bacillus* diet when compared to the controls [28]. The research by Patarapreecha et al. (2018) showed that the addition of *B. bacillus* improved the digestion and utilization of proteins and energy in feed components in the starter and growing periods of pigs [29]. Giang et al. (2011) also demonstrated that pigs fed a diet contain *B. subtilis* had better digestibility of CP [30]. In contrast, several studies reported that the supplementation of bacillus-based probiotics did not affect the DM, E, or nitrogen digestibility in pigs [31,32]. Due to discrepancies in the ages of the animals, the dose of *B. bacillus* species, diet composition, feed form, and interactions with other dietary feed additives, certain disagreements have arisen.

One of the effective ways to reduce the incidence of diarrhea is to promote piglets’ intestinal development by applying some functional feed additives [33,34]. Maruta et al. (1996) reported that the incidence of diarrhea in piglets was significantly decreased, and a low mortality rate was observed when their sows were fed a diet containing *B. subtilis* C-3102 for 6 months [8]. Jang et al. (2009) evaluated probiotics as an alternative in weanling piglets and found that feeding them 0.2% *B. subtilis* tended to improve the diarrhea scores of weanling pigs [35]. Our results also showed that pigs fed a *B. subtilis* C-3102 spore diet had lower diarrhea scores compared to non-supplemented piglets. As reported, the impacts of probiotics appeared to be more viable for younger pigs [36]. This may be the reason why the incidence of diarrhea decreased in weanling piglets by Day 7 in our trial.

The imbalance of intestinal microbiota frequently described around weanling is prone to pathogen invasion and is one of the main underlaying reasons for mortality rate post-weanling. *Bacillus. spp.* helps piglets to establish normal microbiota and eliminate or control potential pathogens in addition to promoting the enhancement and improvement of digestive and immune functions in piglets [37,38]. Cui et al. (2013) also demonstrated that adding *B. subtilis* can manipulate gut microbial communities [39]. Larsen et al. (2014) conducted a principal component analysis (PCA) of pathogen inhibition, and the results showed that *B. subtilis* strains have the potential to efficiently inhibit pathogenic bacteria in the intestine [40]. Similarly with these studies, our results showed that the concentration of *E. coli* tended to decrease, and *Lactobacillus* counts tended to increase when piglets were fed a *B. subtilis* C-3102 spore-supplemented diet. A previous study on the intestinal microbiota of weaned piglets also showed that *E. coli* concentrations increased while the number of *Lactobacillus* decreased after weanling [41]. 

Numerous odorous harmful gases, such as NH_3_, H_2_S, and amines emitted from animal manure in intensive livestock and poultry production, have attracted widespread attention from agricultural experts. These harmful gases not only seriously endanger the health of humans and animals but also cause serious ecological problems [42,43]. However, some researchers have discovered that the harmful gases can be decreased by adding probiotics to feed [32,44,45]. Chen et al. (2006) reported that fecal NH_3_-N was significantly decreased (*p* < 0.05) when pigs were fed diets supplemented with 0.2% bacillus-based probiotics compared to pigs fed basal diets [46]. Wang et al. (2009) reported that the dietary application of *B. subtilis* and *B. licheniformis* could reduce NH_3_ emissions [47]. Similarly, Upadhaya et al. (2015) demonstrated that NH_3_ concentrations appeared to be lower during the growing and finishing phases, and no positive effect was found for other gases such as total mercaptans and H_2_S [45]. Our results showed that the concentration of total fecal mercaptan emissions tended to decrease. In fact, the emission of harmful gases in feces is closely related to the digestive utilization of nutrients in the diet and the intestinal microflora ecosystem [48]. Moreover, Banwart and Brenmer (1975) previously reported that only 0.03% of the total sulfur present in swine manure was volatilized to sulfur gases over a 30-day incubation period [49]. This may be a reasonable explanation for the lack of significant reductions in the H_2_S levels. As well, the lesser difference in NH_3_ may be related with no changes of intestinal microbiota in this study.

## 5. Conclusions

Taken together, our results suggest that dietary supplementation with *B. subtilis* C-3102 spores could benefit the body weight, the average daily gain, and the gain-to-feed ratio of weanling piglets and could improve the ATTD of DM, CP, and E. Additionally, *B. subtilis* supplementation had a significant effect on mercaptan emissions, but not on NH_3_ and H_2_S levels.

## Figures and Tables

**Table 1:** Basal diet composition (as-fed basis) [15].

Ingredients	Phase 1 (Day 1–7)	Phase 2 (Day 8–21)	Phase 3 (Day 22–42)
Extruded corn	37.39	47.90	63.36
Soybean meal (dehulled)	12.00	18.00	28.40
Fermented soybean meal	10.00	8.00	-
Low-temperature fish meal	7.60	2.70	-
Soy oil	3.13	3.20	3.65
Dicalcium phosphate	1.24	1.34	1.36
Limestone	0.60	0.74	-
Sugar	3.00	2.00	2.00
Whey protein	11.00	8.00	-
Lactose	12.80	6.70	-
L-Lysine HCL	0.35	0.46	0.38
DL-Methionine	0.18	0.17	0.16
Threonine	0.21	0.29	0.19
Choline chloride 50%	0.10	0.10	0.10
Vitamin premix ^1^	0.20	0.20	0.20
Mineral premix ^2^	0.20	0.20	0.20
Total	100	100	100
Calculated composition			
Crude protein, %	20.0	19.0	18.0
Crude fat, %	5.40	4.80	4.40
Calcium, %	0.80	0.75	0.70
Phosphorus, %	0.70	0.65	0.60
Digestible energy, MJ/kg	16.7	16.3	15.5
Lysine, %	1.60	1.50	1.30
Methionine, %	0.48	0.45	0.39
Lactose, %	20	12	0

^1^ Provided per kilogram of feed: 20,000 IU of vitamin A; 4000 IU of vitamin D3; 80 IU of vitamin E; 16 mg of vitamin K3; 4 mg of thiamine; 20 mg of riboflavin; 6 mg of pyridoxine; 0.08 mg of vitamin B12; 120 mg of niacin; 50 mg of Ca-pantothenate; 2 mg of folic acid; 0.08 mg of biotin. ^2^ Provided per kilogram of feed: 80 mg of FeSO_4_∙7H_2_O; 140 mg of CuSO_4_∙5H_2_O; 179 mg of ZnO; 12.5 mg of MnO; 0.5 mg of KI; 0.25 mg of CoSO_4_∙7H_2_O; 0.4 mg of Na_2_SeO_3_∙5H_2_O.

**Table 2:** Effects of *B. subtilis* C-3102 spores as probiotics on growth performance in weanling piglets ^1^.

Items	CON	BSC	SEM ^2^	*p*-Value
Body weight, kg				
Day 0	7.53	7.53	0.04	0.943
Day 7	9.01	9.21	0.06	0.068
Day 21	14.6	15.36	0.14	0.022
Day 42	25.72	27.38	0.18	0.003
ADG, g				
Day 0–7	212	240	7	0.044
Day 8–21	400	439	8	0.029
Day 22–42	529	573	7	0.014
Overall	433	473	5	0.004
ADFI, g				
Day 0–7	270	296	7	0.073
Day 8–21	571	603	7	0.027
Day 22–42	835	842	16	0.522
Overall	648	671	7	0.041
G:F				
Day 0–7	0.788	0.813	0.014	0.254
Day 8–21	0.701	0.729	0.015	0.280
Day 22–42	0.641	0.682	0.146	0.120
Overall	0.669	0.705	0.012	0.011

^1^ Abbreviation: CON, basal diet; BSC, CON + 300 g *B. subtilis* C-3102 spores/ton of feed. ^2^ Standard error of means.

**Table 3:** Effects of *B. subtilis* C-3102 spores as probiotics on nutrient digestibility in weanling piglets ^1^.

Items, %	CON	BSC	SEM ^2^	*p*-Value
Day 42				
Dry matter	80.77	82.02	0.33	0.032
Crude protein	77.12	80.66	0.26	<0.0001
Energy	76.73	81.61	0.46	0.0001

^1^ Abbreviation: CON, basal diet; BSC, CON + 300 g *B. subtilis* C-3102 spores/ton of feed. ^2^ Standard error of means.

**Table 4:** Effects of *B. subtilis* C-3102 spores as probiotics on diarrhea scores in weanling piglets ^1^.

Items	CON	BSC	SEM ^2^	*p*-Value
Diarrhea score ^3^				
Day 0	3.69	3.61	0.04	0.230
Day 1–7	3.69	3.54	0.04	0.047
Day 14–21	3.44	3.40	0.03	0.374
Day 35–42	3.40	3.30	0.03	0.110

^1^ Abbreviation: CON, basal diet; BSC, CON + 300 g *B. subtilis* C-3102 spores/ton of feed. ^2^ Standard error of means. ^3^ Diarrhea scores were determined using the following diarrhea scoring system: 1—hard, dry pellet; 2—firm, formed stool; 3—soft, moist stool that retains shape; 4—loose, unformed stool that assumes shape of container; 5—watery liquid that can be poured.

**Table 5:** Effects of *B. subtilis* C-3102 spores as probiotics on microbiota in weanling piglets ^1^.

Items, log^10^ cfu/g	CON	BSC	SEM ^2^	*p*-Value
Day 42				
*E. coli*	5.48	5.36	0.03	0.063
*Lactobacillus*	7.51	7.64	0.04	0.076

^1^ Abbreviation: CON, basal diet; BSC, CON + 300 g *B. subtilis* C-3102 spores/ton of feed. ^2^ Standard error of means.

**Table 6:** Effects of *B. subtilis* C-3102 spores as probiotics on gas emissions in weanling piglets ^1^.

Items, ppm	CON	BSC	SEM ^2^	*p*-Value
Day 42				
Ammonia	7.74	7.46	0.12	0.178
Hydrogen sulfide	5.64	5.32	0.11	0.115
Total mercaptan	3.32	3.14	0.05	0.053

^1^ Abbreviation: CON, basal diet; BSC, CON + 300 g *B. subtilis* C-3102 spores/ton of feed. ^2^ Standard error of means.

## Data Availability

Not applicable.

## Abbreviations

BW	body weight
*B. Subtilis*	*Bacillus subtilis*
ADG	average daily gain
G/F	gain-to-feed ratio
DM	dry matter
CP	crude protein
E	energy
Cr_2_O_3_	chromium oxide
ATTD	apparent total tract digestibility
*E. Coli*	*Escherichia coli*
NH_3_	ammonia
H_2_S	hydrogen sulfide
